# Physical Inactivity in Pulmonary Sarcoidosis

**DOI:** 10.1007/s00408-019-00215-6

**Published:** 2019-03-19

**Authors:** Peter S. P. Cho, Sharmila Vasudevan, Matthew Maddocks, Arietta Spinou, Sarah Chamberlain Mitchell, Claire Wood, Caroline J. Jolley, Surinder S. Birring

**Affiliations:** 1 Centre for Human and Applied Physiological Sciences, School of Basic and Medical Biosciences, King’s College London, London, UK; 2 School of Medicine, King’s College London, London, UK; 3 Cicely Saunders Institute of Palliative Care, Policy and Rehabilitation, King’s College London, Denmark Hill, London, UK; 4 School of Health Sport and Bioscience, University of East London, London, UK; 5 School of Health and Rehabilitation, Keele University, Keele, UK; 6 Department of Respiratory Medicine, King’s College Hospital NHS Foundation Trust, Denmark Hill, SE5 9RS London, UK

**Keywords:** Physical activity, Sarcoidosis, Exercise capacity

## Abstract

**Purpose:**

Reduced physical activity in many chronic diseases is consistently associated with increased morbidity. Little is known about physical activity in sarcoidosis. The aim of this study was to objectively assess physical activity in patients with pulmonary sarcoidosis and investigate its relationship with lung function, exercise capacity, symptom burden, and health status.

**Methods:**

Physical activity was assessed over one week in 15 patients with pulmonary sarcoidosis and 14 age-matched healthy controls with a tri-axial accelerometer (ActivPal™) and the International Physical Activity Questionnaire (IPAQ). All participants underwent pulmonary function tests, 6-min walk test (6MWT) and completed the Fatigue Assessment Scale (FAS), Medical Research Council (MRC) Dyspnoea Scale and the King’s Sarcoidosis Questionnaire (KSQ).

**Results:**

Patients with sarcoidosis had significantly lower daily step counts than healthy controls; mean (SD) 5624 (1875) versus 10,429 (2942) steps (*p* < 0.01) and a trend towards fewer sit-to-stand transitions each day (*p* = 0.095). Only two patients (13%) self-reported undertaking vigorous physical activity (IPAQ) compared to half of healthy individuals (*p* < 0.01). Daily step count was significantly associated with 6MWT distance in sarcoidosis (*r* = 0.634, *p* = 0.01), but not with forced vital capacity (*r* = 0.290), fatigue (*r* = 0.041), dyspnoea (*r* = −0.466) or KSQ health status (*r* = 0.099–0.484). Time spent upright was associated with fatigue (*r* = −0.630, *p* = 0.012) and health status (KSQ Lung scores *r* = 0.524, *p* = 0.045), and there was a significant correlation between the number of sit-to-stand transitions and MRC dyspnoea score (*r* = −0.527, *p* = 0.044).

**Conclusion:**

Physical activity is significantly reduced in sarcoidosis and is associated with reduced functional exercise capacity (6MWD). Fatigue, exertional symptoms and health status were more closely associated with time spent upright and the number of bouts of physical activity, as compared to step counts. Further studies are warranted to identify the factors that determine different physical activity profiles in sarcoidosis.

**Electronic supplementary material:**

The online version of this article (10.1007/s00408-019-00215-6) contains supplementary material, which is available to authorized users.

## Introduction

Sarcoidosis is a multi-system disorder characterised by granulomatous inflammation. The lungs, skin, eyes and the musculoskeletal system are frequently affected [1, 2]. Patients with sarcoidosis often present with dyspnoea, fatigue and arthralgia [3, 4]. The exercise capacity of patients with sarcoidosis has been reported to be reduced, whether assessed with the 6-min walk test (6MWT) or cardiopulmonary exercise test [3–6]. A limitation of measures exercise capacity is that they provide a limited insight into the impact the disease has on activities of daily living [7].

Physical activity is impaired in many chronic diseases and is associated with increased morbidity and mortality [8–11]. Physical activity can be assessed objectively with validated accelerometers for prolonged periods [12, 13]. Recent studies have reported that patients with chronic respiratory disorders such as chronic obstructive pulmonary disease (COPD) [14–16] and idiopathic pulmonary fibrosis (IPF) [8, 9] have reduced physical activity. An important finding of these studies was that outcome measures of lung disease such as lung function, radiological imaging and health-related quality of life do not reflect the physical activity of patients [8, 16, 17]. Bahmer et al. recently reported the clinical predictors of walking physical activity (steps per day) in patients with mild sarcoidosis [18]. The step count was associated with exercise capacity but not with symptoms of physical fatigue [18]. This study, however, did not compare patients to a healthy control group and did not investigate the different types of physical activity in daily life since this was not available with the physical activity monitor they used [18]. There are physical activity monitors available that can measure different types of activity. The ActivPal™, a piezoelectric triaxial accelerometer, can assess daily step count, time spent in an upright posture, stepping time, and the number of sit-to-stand transitions. The reliability and validity of this monitor has been demonstrated in both healthy individuals and patients with COPD [19–22]. The monitor has good reliability for step counts and stepping cadence at different walking speeds in both indoor and outdoor environments and during motor vehicle travel with a measurement error of < 1% [19, 22].

In this pilot study, we aimed to investigate physical activity objectively with a triaxial accelerometer that can assess a range of activity in patients with sarcoidosis and healthy controls and investigate their relationships with exercise capacity, pulmonary function, fatigue and health status.

## Methods

### Participants and Clinical Characterisation

Consecutive participants with pulmonary sarcoidosis were recruited prospectively from a secondary care specialist clinic (King’s College Hospital, London, UK). The diagnosis of sarcoidosis and organ involvement were assessed by a multi-disciplinary team of clinicians and radiologists and established when clinical and histological features were consistent with those described in “A Case-Control Etiologic Study of Sarcoidosis” [23]. Chest radiographs were assessed by an experienced radiologist blinded to clinical details and reported according to the criteria described by Scadding [24]. Inclusion criteria were pulmonary involvement and stable condition in the past 3 months as judged by the clinician. Exclusion criteria were the presence of respiratory conditions other than sarcoidosis, co-morbidities which were considered to affect daily activities (cardiac disease, peripheral vascular disease, stroke, central and peripheral nervous disease other than sarcoidosis, osteoarthritis), upper respiratory tract infection within the last 4 weeks, active psychiatric disorder and the use of walking aids.

Healthy controls were recruited prospectively through advertisement. The healthy control inclusion criteria were normal spirometry and exclusion criteria were identical to those of sarcoidosis subjects. Demographics, anthropometric data and clinical characteristics were recorded. All participants gave informed written consent and the study was approved by the local research ethics committee (NRES Committee London Surrey Borders, 09/H0806/74).

### Physical Activity Monitoring

Physical activity was assessed with the ActivPal™ (PAL Technologies Ltd, Glasgow, UK), a small, lightweight (35 × 53 × 7 mm, 15 g) piezoelectric triaxial accelerometer. The monitor was attached to participants’ upper thigh with hydrogel adhesion pads (PALstickies™, PAL Technologies Ltd, Galsgow, UK) and a Tegaderm™ transparent dressing (3M, St Paul, Minneapolis, USA). Accelerometry measurements were made at a sampling rate of 15 Hz and summarised into 15 s epochs, then downloaded and analysed using the manufacture’s software (ActivPal3™ version 7.1.18, PAL Technologies, Glasgow, UK).

### Subjective Assessments

#### International Physical Activity Questionnaire

Participants were requested to complete the short version of the International Physical Activity Questionnaire (IPAQ) with the reference period being the last 7 days [25]. Craig et al. (2003) demonstrated that the IPAQ has acceptable reliability and validity when compared against accelerometers [25]. The short IPAQ assesses the duration and frequency of moderate (4–8 metabolic equivalents (MET)) and vigorous (> 8 MET) physical activity and the time spent walking in the last 7 days [25]. Activity-specific scores were calculated as per scoring guidelines and were expressed as METs minutes per week and an overall weekly score. A higher score on the IPAQ indicates a higher level of physical activity [25].

#### Fatigue Assessment Scale

The Fatigue Assessment Scale (FAS) is a validated tool to assess fatigue in participants with sarcoidosis [26, 27]. The FAS has 10 items and a score ranging from 10 to 50, where a score greater than 22 indicates fatigue [27].

#### MRC Dyspnoea Scale

Participants were asked to report the impact of breathlessness on their daily activities using the Medical Research Council (MRC) dyspnoea scale (score range 1–5) [28].

#### Health Status: King’s Sarcoidosis Questionnaire

The King’s Sarcoidosis Questionnaire (KSQ) was used to assess the health status of the participants with sarcoidosis [29]. The KSQ is a self-administered questionnaire which has been validated for use in subjects with sarcoidosis and has recently been utilised in clinical trials [29, 30]. The KSQ comprises of 29 questions which are divided into 5 modules: General Health (10 items), Lung (6 items), Skin (3 items), Eye (7 items) and Medications (3 items). The response to each item is recorded on a 7-point Likert scale. Participants completed the General Health and Lung modules and remaining modules as applicable. KSQ Scores range from 0 to 100, where 0 is severe health status impairment and 100 is no impairment [29].

### Lung Function and Exercise Capacity

Spirometry was measured according to the Association for Respiratory Technology and Physiology and the European Respiratory Society guidelines (Jaeger MS-PFT Analyser Unit with Lab Manager Software version 5.32.0) [31, 32]. Participants underwent the 6-min walk test (6MWT) as per the European Respiratory Society (ERS) and American Thoracic Society (ATS) guidelines [33].

### Protocol

Participants completed the questionnaires, spirometry and 6MWT prior to the application of the activity monitor. Participants were instructed to wear the activity monitor for 8 consecutive days commencing on a Monday, removing it briefly only for personal hygiene. An illustrated manual with key information and guide for attaching the activity monitor and training was provided to participants. Participants were given a diary to record times spent sleeping and when the activity monitor was detached. Activity monitor data from the first and final day was excluded in the analysis since this included activity related to the study hospital visits. Therefore, activity data from 6 days, which included 4 weekdays and 2 weekend days were analysed. When the activity monitor was attached for < 8 h, data for that day were discarded.

### Statistical Analysis

Data were analysed using Prism® Version 7.0c for macOS version 10.13.3 (GraphPad Software; San Diego, California, USA). The distribution of data was assessed using the D’Agostino and Pearson test. Parametric data were expressed as mean (standard deviation, SD) whereas non-parametric data were expressed as median (interquartile range, IQR). Parametrically distributed data were analysed with independent unpaired Welch’s *t* tests to compare sample means, whereas comparison of non-parametric data was carried out using the Mann–Whitney *U* test. Chi-squared test was utilised for categorical data. Correlations between variables were analysed with Pearson’s correlation coefficient (*r*) for parametric data and Spearman’s correlation coefficient (*ρ*) for non-parametric data. A *p* value of less than 0.05 was considered statistically significant.

A formal sample size was not calculated since this was a pilot study. A recent study investigating physical activity was able to detect significantly reduced physical activity in 15 participants with fibrotic idiopathic interstitial pneumonia [8].

## Results

### Participant Characteristics

Fifteen consecutive participants with pulmonary sarcoidosis and fourteen healthy controls were recruited for this study. The demographics and clinical characteristics are shown in Table 1. Healthy controls were matched for age, gender and body mass index (BMI) (Table 1). One participant with sarcoidosis (active cardiac disease) and one healthy control (FEV_1_ < 60% predicted) were excluded. A greater proportion of participants with sarcoidosis were of black Afro-Caribbean ethnicity compared to controls (Table 1). No participants had a clinical diagnosis of anxiety or depression.

**Table 1:** Demographic and clinical characteristics of the study participants

	Healthy controls (*n* = 14)	Sarcoidosis (*n* = 15)
Age (years)	46.5 (5.5)	52.7 (15.5)
Female (*n*, %)	10 (71)	11 (73)
BMI (kg/m^ 2^)	27.1 (4.1)	27.7 (4.4)
Ethnicity (*n*, %)		
Caucasian	10 (71)	3 (20)
Afro-Caribbean	1 (7)	9 (60)
Other	3 (21)	3 (20)
Smoking status (*n*, %)		
Current	0 (0)	1 (7)
Ex-smoker	4 (29)	4 (27)
Never	10 (71)	10 (67)
Time since diagnosis (years)		8.3 (8.4)
Organ involvement	N/A	
Lungs		15 (100)
Skin		6 (40)
Eyes		3 (20)
Other		8 (53)
Multiple organ involvement	N/A	
≥2		12 (80)
≥3		5 (33)
Scadding stage	N/A	
0		0 (0)
1		4 (27)
2		3 (20)
3		4 (27)
4		4 (27)
Pulmonary function		
FEV_1_% predicted	98.1 (14.7)	65.7 (18.6)*
FVC% predicted	107.3 (17.0)	78.5 (17.7)*
FEV_1_/FVC	75.9 (6.1)	69.1 (12.6)*
*T*_LCO_% predicted	86.8 (7.0)	58.4 (13.8)*
ACE (IU/L)	N/A	54 (28)
Immunosuppressant	N/A	
None		5 (33)
Prednisolone		10 (67)
Methotrexate		3 (20)
Azathioprine		1 (7)
Hydroxychloroquine		2 (14)
Inhaled corticosteroid		2 (14)
MRC dyspnoea score	1 (1)	3 (1)
FAS	15 (4)	23 (7)
KSQ	N/A	
General Health		55 (20)
Lung		60 (19)
Medication		68 (33)
General Health + Lung		57 (20)

Data presented as mean (SD) or absolute number (%). Immunosuppressants were those prescribed at the time of the study

*BMI* body mass index; *MRC* Medical Research Council; *ACE* angiotensin-converting enzyme; *FEV*_*1*_ forced expiratory volume in 1 s; *FVC* forced vital capacity; *T*_*LCO*_ diffusion capacity of lung for carbon monoxide; *FAS* Fatigue Assessment Scale; *KSQ* King’s Sarcoidosis Questionnaire

*p < 0.05

### Physical Activity Monitoring

**Fig. 1:**
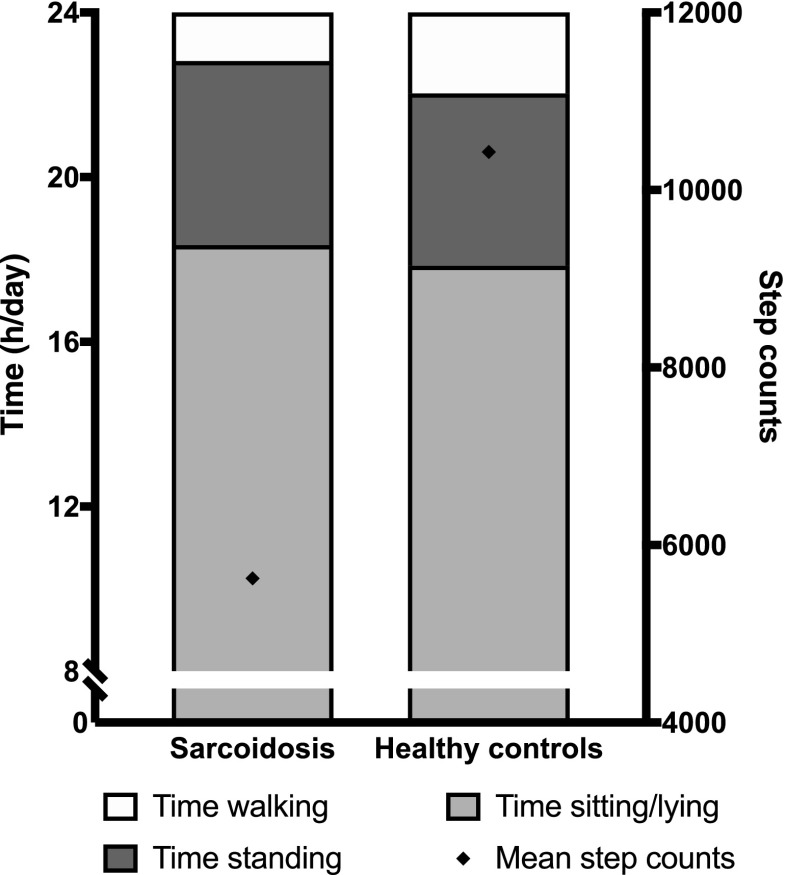
Physical activity in participants with sarcoidosis and healthy controls

Participants with sarcoidosis reported they were adherent with objective physical activity monitoring; only one participant (healthy control) reported non-adherence for more than 8 h for 2 days. Data from these 2 days for this participant was not included in the analysis. Physical activity data are summarised in Fig. 1.

#### Step Count and Stepping Time

**Fig. 2:**
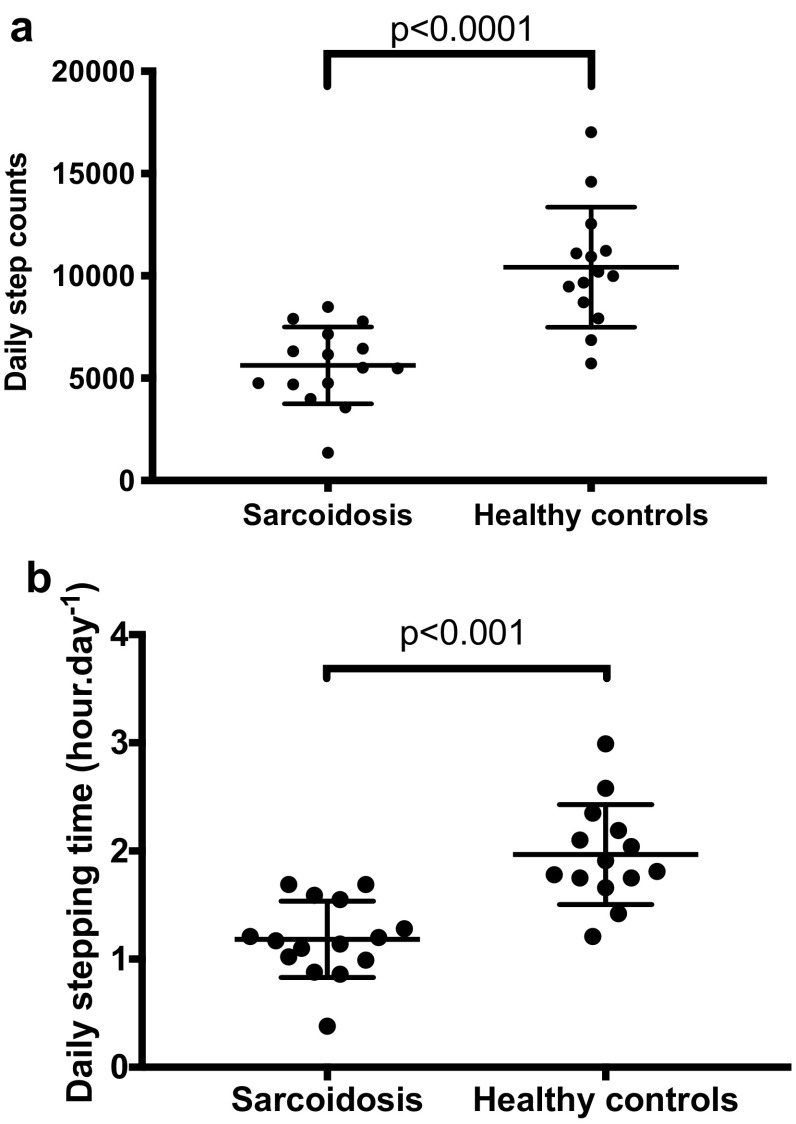
A comparison of physical activity (steps) between participants with sarcoidosis and healthy controls on **a** daily step counts and **b** daily stepping time

Daily step counts were significantly lower in participants with sarcoidosis as compared to healthy controls; mean (SD) 5624 (1875) versus 10,429 (29,422) steps, respectively, mean difference (95% CI) 4804 (2888–6720) steps, *p* < 0.001 (Fig. 2a; Table 2). Daily stepping time was also significantly lower in participants with sarcoidosis than in healthy controls; mean (SD) 1.18 (0.35) versus 1.97 (0.46) h/day, respectively, mean difference (95% CI) 0.78 (0.47–1.10), *p* < 0.001 (Fig. 2b; Table 2).

**Table 2:** Objective and subjective assessments of physical activity in participants with sarcoidosis and healthy controls

	Healthy controls (*n* = 14)	Sarcoidosis (*n* = 15)
Objective assessments		
6MWD (m)	487 (92)	375 (59)*
Physical activity		
Step counts (/day)	10,429 (2942)	5624 (1875)^†^
Sit to stand transitions (/day)	63 (47–69)	53 (35–59)
Upright time (h/day)	5.69 (5.26–6.47)	5.71 (3.73–7.56)
Stepping time (h/day)	1.86 (1.73–2.23)	1.17 (0.99–1.55)^†^
Standing time (h/day)	3.77 (3.48–4.60)	4.16 (2.86–6.27)
Subjective assessments		
IPAQ scores		
Overall (MET-min/week)	3230 (1912–5599)	2153 (1152–4925)
Walking (MET-min/week)	1287 (412–1914)	1386 (330–3366)
Moderate PA (MET-min/week)	840 (210–1510)	320 (127–2175)
Vigorous PA (MET-min/week)	480 (0–3360)	0 (0–0)^‡^

Data presented as mean (SD) or median (IQR)

*6MWD* 6-min walk distance; *IPAQ* International Physical Activity Questionnaire; *MET* metabolic equivalent

*p = 0.0009

^†^*p* < 0.0001

^‡^*p* = 0.017

#### Sit-to-Stand Transitions and Standing Time

There was a trend towards a lower number of sit-to-stand transitions in participants with sarcoidosis than in healthy controls; median (IQR) 53.0 (35.0–59.0) versus 62.5 (46.8–68.8) transitions/day, respectively, *p* = 0.095. There was no significant difference in the time spent standing between participants with sarcoidosis and healthy controls; median (IQR) of 4.16 (2.86–6.27) versus 3.77 (3.48–4.60) h/day respectively, *p* = 0.915 (Table 2).

#### Association Between Physical Activity and Clinical Characteristics

There was no significant association between mean daily step count and age (*r* = − 0.43, *p* = 0.107), BMI (*r* = 0.06, *p* = 0.842), Scadding Chest X-ray staging (*ρ* = − 0.35, *p* = 0.202), number of organs involved (*ρ* = 0.23, *p* = 0.410) or use of corticosteroids (*p* = 0.17). There was a trend towards an association between number of organs involved and daily standing time (*ρ* = − 0.47, *p* = 0.082).

#### Exercise Capacity (6MWT)

**Fig. 3:**
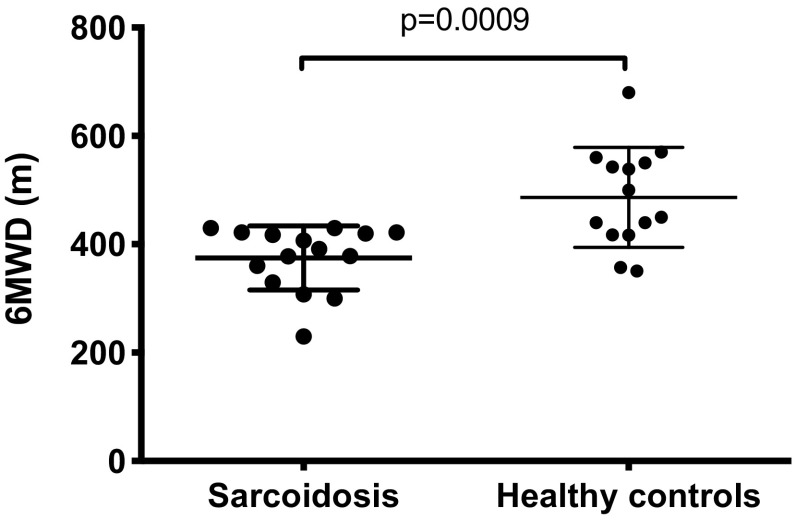
A comparison of exercise capacity between participants with sarcoidosis and healthy controls

The 6-min walking distance (6MWD) was significantly lower in participants with sarcoidosis compared to healthy controls; mean (SD) 375 (59) versus 487 (92) metres; mean difference (95% CI) 112 (52, 172) metres, *p* < 0.001 (Fig. 3). There was a significant correlation between 6MWD and the daily step count in participants with sarcoidosis (*r* = 0.63, *p* = 0.011; Fig. E1). There was a significant association between 6MWD and self-reported vigorous activity on IPAQ (*ρ* = 0.52, *p* = 0.048) and a trend to significance for the relationship between 6MWD and self-reported walking activity on IPAQ (*ρ* = 0.48, *p* = 0.071). There was no significant correlation between 6MWD and self-reported total physical activity on IPAQ (*ρ* = 0.06, *p* = 0.829).

### Lung Function

There was no association between daily step count and lung function measures in participants with sarcoidosis (FEV_1_: *r* = 0.27, *p* = 0.330; FVC: *r* = 0.29, *p* = 0.294; T_LCO_: *r* = − 0.01, *p* = 0.963). This was in contrast to healthy participants in which there were significant relationships (FEV_1_: *r* = 0.71, *p* = 0.007; FVC: *r* = 0.69, *p* = 0.009; *T*_LCO_: *r* = 0.80, *p* = 0.001). There was no significant association between lung function measures and daily stepping time in participants with sarcoidosis (FEV_1_: *r* = 0.17; FVC: *r* = 0.30; T_LCO_: *r* = − 0.11), daily standing time (FEV_1_: *r* = − 0.02; FVC: *r* = − 0.18; *T*_LCO_: *r* = − 0.31) or daily sit to stand transitions (FEV_1_: *r* = − 0.17; FVC: *r* = − 0.05; T_LCO_: *r* = − 0.03), all *p* > 0.05.

### Subjective Assessments

#### International Physical Activity Questionnaire

Vigorous physical activity was significantly lower in participants with sarcoidosis than in healthy controls; median (IQR) of 0 (0) versus 480 (0–3360) MET-min/week, respectively, *p* = 0.017 (Table 2). The majority of participants with sarcoidosis, 13 (87%), reported no vigorous activity in contrast to 7 (50%) healthy controls (*p* < 0.01). Participants with sarcoidosis reported lower overall physical activity compared to healthy controls but this was not statistically significantly different; median (IQR) 2153 (1152–4925) versus 3230 (1912–5599) MET-min/week, respectively, *p* = 0.326 (Table 2).

In participants with sarcoidosis, there was a significant association between walking activity on IPAQ and daily stepping time (*ρ* = 0.61, *p* = 0.018). There was a trend to significance for the relationship between walking activity (IPAQ) and daily step count (*ρ* = 0.52, *p* = 0.052). There was no association between IPAQ total score and objective daily step count (*ρ* = 0.16, *p* = 0.594).

#### Fatigue: Fatigue Assessment Scale

Participants with sarcoidosis reported more fatigue than healthy controls; mean (SD) FAS score 23.27 (6.70) versus 15.43 (3.65); mean difference (95% CI) 7.84 (3.72–11.96), *p* < 0.001 (Table E1). There was a significant relationship between fatigue (FAS) in sarcoidosis and the time spent upright per day measured with the accelerometer (*r* = − 0.63, *p* = 0.013) and time spent standing per day (*r* = − 0.67, *p* = 0.006; Fig. E2a). There was no significant association between fatigue and sit to stand transitions in participants with sarcoidosis (*r* = − 0.01, *p* = 0.973) or daily step counts (*r* = 0.04, *p* = 0.884). Fatigue was not associated with self-reported overall IPAQ physical activity (*ρ* = 0.28, *p* = 0.325) in participants with sarcoidosis (Table E1). For healthy controls, there was no significant correlation between fatigue and objective assessments of physical activity (ρ = − 0.03 to 0.15, all p > 0.05).

#### MRC Dyspnoea

Participants with sarcoidosis reported mean (SD) MRC dyspnoea score 3.0 (1). There was a significant correlation between MRC dyspnoea scores and the number of sit to stand transitions (*r* = − 0.53, *p* = 0.044). There was also a trend towards significance in the relationship between the MRC dyspnoea scores and daily step counts in participants with sarcoidosis (*r* = − 0.47, *p* = 0.080).

#### Health Status: King’s Sarcoidosis Questionnaire

The health status of the participants with sarcoidosis measured with the KSQ was moderate to severely impaired (Table 1). There were significant correlations between the KSQ Lung scores and time spent standing per day (*r* = 0.58, *p* = 0.023; Fig. E2b) and time spent upright (*r* = 0.52, *p* = 0.045). There was no significant association between KSQ Lung scores and daily step count (*p* = 0.484), daily stepping time (*p* = 0.611) and sit to stand transitions (*p* = 0.850). There was no significant correlation between KSQ General Health scores and objective measures of physical activity (*r* = − 0.38 to 0.42, *p* = 0.115–0.262). There was a significant association between the self-reported overall physical activity (IPAQ scores) and the KSQ lung domain (ρ = − 0.56, *p* = 0.042; Table E1).

## Discussion

This is the first study to objectively assess physical activity in sarcoidosis with a validated triaxial accelerometer [19–22]. Physical activity was significantly reduced in patients with sarcoidosis compared to healthy controls. The 6-min walk test had the strongest association with physical activity in patients with sarcoidosis. Surprisingly, there was no significant association between daily step counts and fatigue. Fatigue was, however, associated with reduced time spent upright. There was no association between lung function measures and physical activity in sarcoidosis.

We investigated different types of physical activity in sarcoidosis made possible by utilising a triaxial accelerometer [19–22]. Daily step counts and daily stepping time were reduced in patients with sarcoidosis compared with healthy controls. Participants with sarcoidosis spent less time walking and had a lower cadence (steps/minute) compared to healthy controls. Self-reported physical activity was lower in patients with sarcoidosis compared with healthy controls, but the difference was not statistically significant. Self-assessed physical activity is known to correlate poorly with objective measures [34]. Self-reported vigorous physical activity was, however, significantly reduced in patients with sarcoidosis. Furthermore, objective daily step counts correlated better with self-reported IPAQ walking activity compared to IPAQ overall activity which reflects a wide range of daily activities, many of which do not involve walking. The 6MWT had the strongest association with physical activity of all clinical outcome measures assessed, similar to findings in patients with interstitial lung disease and sarcoidosis [8, 9, 18]. The correlation between 6MWD and physical activity was moderate, suggesting that measures of exercise capacity do not fully capture physical activity. There was a trend towards an association between dyspnoea and daily step counts and sit-to-stand transitions. There was, however, no association between daily step counts and lung function or health-related quality of life. Health-related quality of life and dyspnoea were, however, associated with reduced time spent upright.

Fatigue is a common and troublesome symptom of sarcoidosis [6, 35, 36]. We found no association between daily step counts and fatigue, consistent with the findings of Bahmer et al. [18]. However, fatigue was associated with reduced time spent upright, and therefore, the impact of fatigue may be limited to specific types of physical activity. The reason for reduced physical activity in sarcoidosis is unclear. Physical activity parameters were not related to age, BMI, disease severity or medication use. The lack of clear associations with individual factors suggests that there may be multi-factorial reasons for reduced physical activity. The factors potentially relevant include cardiac function, psychological and medications [17, 37, 38]. The mechanisms of reduced physical activity in sarcoidosis should be studied further.

There are some limitations to our study. We investigated a small number of patients, and therefore, our findings need to be replicated in a larger study. Physical activity was assessed over 1 week, and this interval may have been too short to be representative of habitual physical activity. Some patients were taking treatments such as corticosteroids though it has been demonstrated that the use of corticosteroids does not affect muscle strength [4]. We did not find a significant difference in physical activity between those patients taking corticosteroids and no medications. There were differences in ethnicity between patients and healthy controls which may have biased our results. Our sample size was too small to analyse for any differences between ethnic groups. We did not assess the level of education and occupation of subjects and its impact on activity. Marshall et al. demonstrated that social class moderates the relationship between ethnicity and physical activity, and suggested that a focus on ethnicity in physical activity detracts from the significant role played by social classes [39]. It is possible that the control group may have been motivated to participate in an activity study and, therefore, were physically fit. Future studies using a matched case–control design can overcome this potential bias.

In conclusion, physical activity is reduced in patients with sarcoidosis compared with healthy controls. The assessment of physical activity was well tolerated. Physical activity monitors can provide a unique assessment of the impact of disease on function that is not captured by existing clinical outcome measures for sarcoidosis, and can potentially be used to assess response to therapy. The mechanism for reduced physical activity in sarcoidosis is unclear but is likely to be multi-factorial and needs further study.

## Electronic supplementary material

Below is the link to the electronic supplementary material.

Supplementary material 1 (DOCX 12 KB)

Supplementary material 2 (EPS 161 KB)

Supplementary material 3 (EPS 238 KB)
