# Supplementary material for: Physical Inactivity in Pulmonary Sarcoidosis
Source: Lung. 2019 Mar 19;197(3):285–93. doi: 10.1007/s00408-019-00215-6 (PMC6520325; doi:10.1007/s00408-019-00215-6)
Supplement: Supplementary file 1 — Supplementary material 1 (DOCX 12 KB) [file 408_2019_215_MOESM1_ESM.docx]

**Table E1.** The relationship between physical activity, fatigue and quality of life in participants with sarcoidosis.

|  | **Correlation coefficient** | **p-value** |
| --- | --- | --- |
|  | **Subjective physical activity (overall IPAQ)^*^** | |
| FAS | 0.283 | 0.325 |
| KSQ |  |  |
| General Health | -0.491 | 0.077 |
| Lung | -0.555 | 0.042 |
| General Health + Lung | -0.407 | 0.148 |
|  | **Objective physical activity (mean daily step counts)** ^†^ | |
| FAS | 0.041 | 0.884 |
| KSQ |  |  |
| General Health | -0.384 | 0.157 |
| Lung | -0.196 | 0.484 |
| General Health + Lung | -0.443 | 0.099 |

IPAQ = International Physical Activity Questionnaire; FAS = Fatigue Assessment Scale; KSQ = King’s Sarcoidosis Questionnaire

^*^Spearman’s rank correlation. ^†^Pearson’s correlation
